# Optimizing Wrist Splint Fitting Parameters Through Artificial Intelligence Analysis

**DOI:** 10.7759/cureus.71726

**Published:** 2024-10-17

**Authors:** Ashkan Sedigh, Meysam Fathi, Peter K Beredjiklian, Amir Kachooei, Michael Rivlin

**Affiliations:** 1 Orthopedics, Department of Orthopedic Surgery, Division of Hand Surgery, Rothman Orthopedic Institute, Jefferson Medical College, Philadelphia, USA; 2 Hand Surgery, Rothman Orthopedic Institute, Philadelphia, USA; 3 Orthopaedic Surgery, Rothman Orthopedic Institute, Philadelphia, USA; 4 Orthopaedic Surgery, Rothman Orthopedic Institute, Orlando, USA; 5 Division of Hand Surgery, Rothman Orthopedic Institute, Philadelphia, USA

**Keywords:** 3d printing, 3d scanning, artificial intelligence, brace, machine learning, wrist

## Abstract

Introduction

The current method for determining the appropriate wrist splint size in the clinical setting relies on measuring wrist circumference, but this approach often fails to ensure optimal fit. This study evaluates additional hand features using 3-dimensional (3D) scanned data and Artificial Intelligence (AI) to improve the fit of pre-fabricated wrist splints. We hypothesize that wrist and forearm widths can provide a more accurate fitting than wrist circumference alone.

Materials and methods

We recruited 54 healthy volunteers to be scanned. Each volunteer was fitted with a standard wrist brace (Short Arm Brace, Ossur, Iceland), and 3D data from their hands were collected using an infrared-based 3D scanner (Einscan Pro, Shining3D, China). The 3D scanned data were then analyzed to identify and measure 14 distinct hand features. To explore the relationship between these hand features and the optimal splint size, we generated a categorical correlation map. This map identified hand features that were most strongly correlated with splint size categories (small, medium, large). Subsequently, we developed a classification algorithm to predict the appropriate splint size based on the correlated hand features. We utilized three different machine learning models for this purpose: Extreme Gradient Boosting (XGB) Classifier, RandomForestClassifier, and Support Vector Classifier (SVC). Each of these classifiers was trained and evaluated to determine their accuracy and effectiveness in predicting the correct splint size.

Results

Wrist width showed the highest classification accuracy (91%) for both the XGB Classifier and RandomForestClassifier. The measurements including hand wrist width, mid-forearm width, and hand crease line width also performed well with the XGB Classifier, achieving an accuracy of 90%. The SVC showed consistent performance across various feature sets, with the highest accuracy of 81% for the measurements. Overall, these findings suggest that wrist width is the most predictive feature for splint size classification, with additional features providing minimal enhancement.

Conclusions

Artificial intelligence, combined with 3D scanning, can accurately predict wrist splint size from a single image acquisition, enabling contactless, personalized fitting. This approach can improve patient outcomes by enhancing the fit of prefabricated splints.

## Introduction

Prefabricated wrist braces are commonly available and may be as effective as custom-made braces [1-4]. An optimal splint fit is essential to maximizing pain and function. In contrast, poorly fitted splints negatively impact compliance and clinical effectiveness [5-7]. With increases in the utilization of telemedicine for the delivery of care, remote fitting of braces for orthopedic conditions is likely to become more prevalent. Despite its widespread use, there is still no standard approach to determining the appropriate size of prefabricated splints.

The manufacturer’s sizing guidelines do not always guarantee an optimal fit [7]. Recent research demonstrates substantial advances in artificial intelligence (AI) and machine learning (ML) for optimizing wrist splint design and fit [8]. Machine learning, a branch of artificial intelligence, uses trained algorithms to predict outcomes based on the input data [9,10]. ML algorithms enhance accuracy and reduce human-based errors [11,12].

The goals of this study are (1) to identify which hand and wrist features are most effective in determining the best-fitting splint size, and (2) to determine if there are any other hand and wrist features that can more accurately determine the size of the best-fitting prefabricated splint.

## Materials and methods

Study design and participants

**Figure 1:**
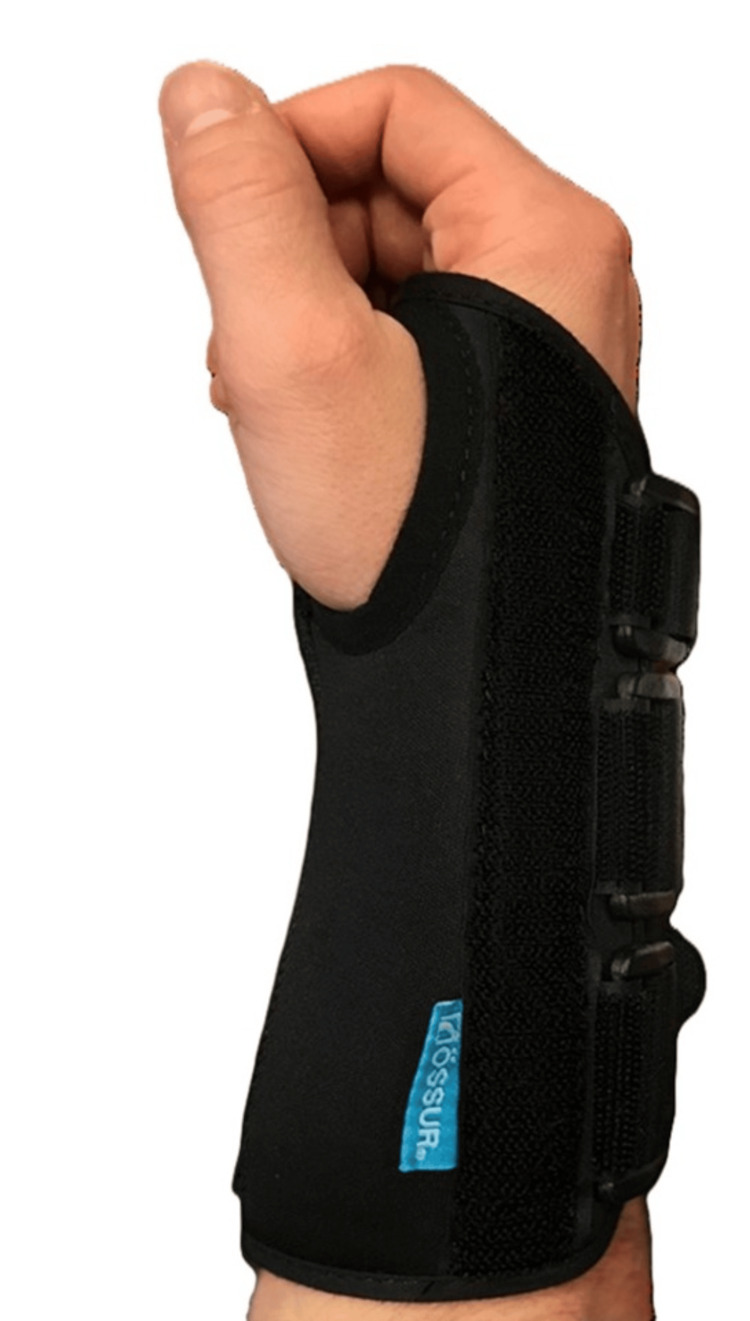
Prefabricated wrist brace. Figure courtesy of Ashkan Sedigh (lead author).

This study involved recruiting healthy volunteers aged 18 or older without a history of previous hand surgery to create a dataset for developing a machine learning-based splint size prediction model. A total of 54 hands were scanned using a 3D scanner from volunteers with no history of hand deformities, trauma, or surgeries that could affect normal hand anatomy. All participants provided informed consent, and ethical approval was obtained from the institution’s review board (IRB Control # 13D.432). To ensure uniformity in data collection, all participants were fitted with a standard wrist brace (Short Arm Brace, Ossur, Iceland) before scanning (Figure 1).

The subjects independently selected the best brace fit from an array of available braces in person (small, medium, large). Each participant's hand was scanned in a neutral position, ensuring consistency in the captured images.

3D scanning and data acquisition

Hand data were captured using a high-resolution, infrared-based 3D scanner (Einscan Pro, Shining3D, China). The scanner operates by projecting infrared light onto the surface of the hand and measuring the reflection to generate a three-dimensional image with precise geometric details. The scanner was recalibrated before each scanning session to ensure the accuracy and reliability of the data.

Each hand was scanned three times to reduce the risk of errors or noise in the data. Any outlier scans with excessive noise or distortion were excluded from the final dataset. The final set of 54 high-quality 3D scans was used for feature extraction and further analysis. All scans were stored in a standardized digital format for compatibility with analysis software (Blender, v. 3.4, USA).

Hand feature extraction

**Figure 2:**
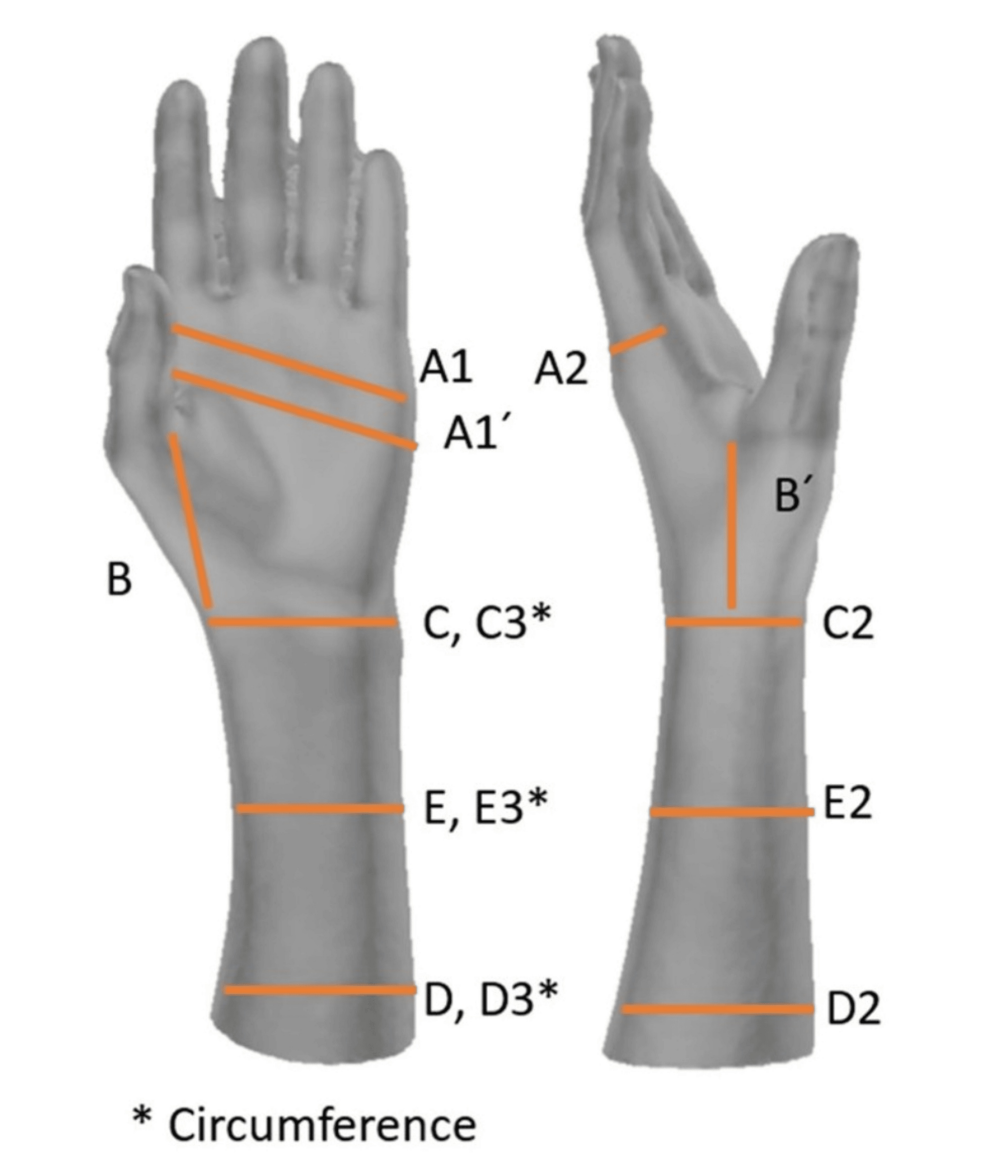
Measured features on the 3D-scanned hand. Wrist crease width (C); Wrist crease thickness (C2); Wrist circumference (C3); Distal forearm (D); Distal forearm thickness (D2); Distal forearm circumference (D3); Mid-forearm (E); Mid-forearm thickness (E2); Mid-forearm circumference (E3); Distal palmar crease (A1); proximal palmar crease (A1 prime); distal palmar crease thickness (A2); distance from the first web space to the wrist crease (B); distance from the first web space to the dorsal continuation of the palmar wrist crease (B prime). Figure courtesy of Ashkan Sedigh (lead author).

The 3D scans were analyzed using commercially available software (Blender v. 3.4, USA), which allowed for precise measurement of the hand's anatomical features. A total of 14 distinct features were identified and extracted from each hand scan (Figure 2).

Included feature measurements were the wrist crease width (C), wrist crease thickness (C2), wrist circumference (C3), distal forearm (D), distal forearm thickness (D2), distal forearm circumference (D3), mid-forearm (E), mid-forearm thickness (E2), mid-forearm circumference (E3), distal palmar crease (A1), proximal palmar crease (A1 prime), distal palmar crease thickness (A2), the distance from the first web space to the wrist crease (B), and the distance from the first web space to the dorsal continuation of the palmar wrist crease (B prime). All measurements were performed in millimeters.

These features were selected based on their relevance to wrist brace fitting and the expected variability across different hand sizes. Measurements were performed manually within the software (Blender v.3.4, USA) to ensure the highest accuracy possible. All measurements were performed manually for each parameter for all 54 hands.

Once extracted, the measurements were exported as a numerical dataset for further analysis. The goal was to understand how these features correlate with the appropriate splint size and to use this information for machine learning classification.

Categorical correlation analysis

A categorical correlation analysis was performed to determine the relationship between the extracted hand features and splint size categories (small, medium, and large). Specifically, we used Spearman's rank correlation coefficient to assess the strength and direction of the correlation between each hand feature and the splint size category.

A categorical correlation map was generated, visually representing the relationship between each hand feature and the splint sizes. Features with the highest correlation to splint size were identified as key predictors for the subsequent machine-learning models. This analysis helped to filter out irrelevant or weakly correlated features, allowing the models to focus on the most significant predictors.

Machine learning models and classification

To build a predictive model for splint size based on hand features, we employed three machine learning classifiers using our developed Python code: Extreme Gradient Boosting (XGB) Classifier, RandomForestClassifier (RFC), and Support Vector Classifier (SVC). These classifiers were selected based on their well-established performance in classification tasks and their ability to handle multi-feature data.

The goal was to predict the correct splint size (small, medium, or large) for each hand based on the 14 extracted features. Each model was trained to recognize patterns in the feature data and classify the hand into one of the three size categories. After training, the models were evaluated on the test set. Several performance metrics were used to assess each model's effectiveness:

Accuracy: The proportion of correct predictions across all test cases; Recall: The proportion of true positive predictions among all actual positives (i.e., the ability to detect all instances of a particular splint size); F1-score: The harmonic mean of precision and recall, providing a single metric to balance both.

## Results

The machine learning models were evaluated based on their ability to predict the correct splint size (small, medium, large) using the hand features extracted from the 3D scans. The performance of the three classifiers, XGB, RFC, and SVC, was compared using accuracy as the primary performance metric. A categorical correlation map was generated, visually representing the relationship between each hand feature and the splint sizes (Table 1).

**Table 1:** Correlation of each measured wrist feature with splint size Correlation indicates Spearman correlation coefficient. p-value is considered significant at p<0.05.

Feature	Correlation	p-value
A1	0.51	<0.001
A1'	0.51	<0.001
A2	0.16	0.02
B	0.18	0.02
B'	0.19	0.002
C	0.65	<0.001
C2	0.22	0.001
C3	0.45	<0.001
D	0.13	0.003
D2	0.39	0.003
D3	0.29	0.003
E	0.60	<0.001
E2	0.34	0.01
E3	0.41	0.002

Features with the highest correlation to splint size were identified as key predictors for the subsequent machine-learning models.

Classification accuracy of individual features

The feature representing hand wrist width (C) emerged as the most predictive for splint size classification across all models. Both the XGB Classifier and RFC achieved the highest classification accuracy of 91% when using only this feature. This indicates that wrist crease width alone is a strong predictor for determining the appropriate splint size.

In contrast, the SVC classifier exhibited consistent but lower performance. The highest accuracy achieved by SVC was 81%, observed when using various feature sets, including the wrist crease width (C). Despite lower accuracy compared to XGB and RFC, SVC demonstrated stable performance across different combinations of features (Table 2).

**Table 2:** Recall, F1 score and Accuracy for feature set C XGB: Extreme Gradient Boosting; SVC: Support Vector Classifier.  Data represented as a percentage.

Machine Learning Classifiers	Recall	F1 Score	Accuracy
RandomForestClassifier	80%	82%	82%
XGB Classifier	91%	95%	91%
SVC	91%	95%	91%

Performance with multiple features

When additional features were included in the model, specifically, mid-forearm width (E) and hand crease line width (A1), the XGB Classifier maintained an accuracy of 90%, which is nearly identical to the performance with the wrist width (C) alone. This suggests that while these additional features provide some marginal value, they do not significantly enhance the model's performance over using wrist width alone. The RFC also performed similarly, with no noticeable improvement in accuracy when additional features were added, supporting the observation that wrist width (C) is the most influential feature for classification. The recall, accuracy, and F1 scores are reported in Table 3 for three different classification-tested algorithms (Table 3). 

**Table 3:** Classifier accuracy Comparison of classifiers with each parameter set. XGB: Extreme Gradient Boosting; SVC: Support Vector Classifier. Data is represented as a percentage.

Hand/wrist feature	XGB Classifier Accuracy (%)	RandomForestClassifier Accuracy (%)	SVC Accuracy (%)
C, E and A1	91%	82%	82%
C	91%	91%	82%
C and E	82%	82%	82%
E	73%	64%	82%
A1	54%	45%	73%

## Discussion

AI is rapidly evolving in all aspects of digital life, and researchers are trying to use it to reduce human decision-making errors [12]. Using artificial intelligence classifiers, we found that wrist width has the highest accuracy for determining the size of a prefabricated splint among 14 hand and wrist features. This measurement showed a 91% classification accuracy for both the XGB and RFC classifiers. Mid-forearm width and hand crease line width also performed well with the XGB Classifier, achieving an accuracy of 90%. The SVC showed consistent performance across various measurement sets, with the highest accuracy of 81% for the measurements. 

There is a lack of research regarding determining the correct prefabricated splint size. To our knowledge, this is the first study in the literature to evaluate AI methodologies in the application of orthotic sizing technologies in the clinical setting. The typical practice is determining the size based on wrist circumference measurement with a tape. Improper fit is a usual complaint of patients [13]. It is recommended that the use of prefabricated splints should always be combined with an accurate measurement of the forearm and hand for a better fit. However, no study in the literature has determined the best sizing method [2]. We compared 14 features and found that considering forearm and hand measurements in addition to wrist width provides minimal enhancement. We believe prefabricated wrist splints should be selected based on the wrist width, as the analysis showed a 91% accuracy for determining the size with accuracy compared to a lower accuracy with the current sizing method [12].

Moreover, the current sizing method, which relies on measuring wrist width, has demonstrated only a moderate correlation with splint size. Notably, wrist crease width (C) exhibits the highest correlation and may serve as a more accurate predictor for determining optimal splint size. This suggests that wrist crease width could be adopted as a superior metric for sizing purposes, improving the precision of splint fitting.

Promoting telemedicine is an important feature of this study. Telemedicine eases access to medical care remotely, reduces the risk of contagious diseases, and makes medical services more cost-effective or accessible [14]. Using wrist width measurement allows the patients to order the best-fitting splint online based on a calibrated photograph of their hand without needing in-person visits. This saves time and money for both patients and the healthcare system. In other words, a potential application of this technology would be to garner the ability to fit an orthotic remotely by simply having a patient upload a photograph of their extremity. 

Changes in the patient’s body posture, variations in tape pressure during measurement, and the identification of reference points are some downsides of manual measurements [15-18]. The use of three-dimensional scanning is becoming more common in the healthcare system. It is considered more accurate, reliable, and reproducible than historically measuring the body parts with calipers and tapes [15,17]. One of the strengths of our study is the use of three-dimensional hand data obtained through an infrared scanner, which allowed us to accurately and reliably measure the circumferential and non-circumferential features of the hand. Our findings revealed that wrist width has the highest correlation with optimal splint sizing. Therefore, the sizing process can be simplified by using a tape measure to measure wrist width alone to determine the best splint size.

Future research could address some of our limitations. Our study group included healthy individuals without hand or wrist conditions, but the efficacy of machine learning predictions may differ in patients with specific hand or wrist issues. We could not validate our findings in a clinical setting nor evaluate their outcome on pain, comfort, and functional enhancement. Moreover, we have only tested the algorithm on three different sizes, whereas the XXS, XS, and XL can be part of the measurements. No children were included in the study, as their measurements have much greater variability.

## Conclusions

Wrist width has the highest predictive accuracy for determining the appropriate size of prefabricated splints. Utilizing this metric to optimize the splint fitting may enhance compliance and improve the outcomes. Additionally, wrist width measurement offers a novel approach to remote splint fitting within the context of telemedicine, reducing costs and increasing access to orthotics. This study can be a foundation for further research on validating wrist width sizing in the clinical setting and improving sizing methods for other wearable medical devices. 
